# A genetic transformation system for the heterotrophic diatom *Nitzschia putrida* (Bacillariophyceae)

**DOI:** 10.1111/jpy.70070

**Published:** 2025-08-14

**Authors:** Longji Deng, Yixuan Li, Yasuhiro Tanizawa, Yasukazu Nakamura, Ryoma Kamikawa, Amanda Hopes, Thomas Mock

**Affiliations:** ^1^ School of Environmental Sciences University of East Anglia, Norwich Research Park Norwich UK; ^2^ Department of Informatics, National Institute of Genetics Research Organization of Information and Systems Shizuoka Japan; ^3^ Graduate School of Agriculture Kyoto University Kyoto Japan

**Keywords:** diatom, gene expression, molecular

## Abstract

Diatoms are important primary producers in aquatic ecosystems. Most of them are photoautotrophs and have evolved to thrive under diverse environmental conditions from the poles to the tropics. However, some diatom species such as *Nitzschia putrida* have lost photosynthesis and have therefore become free‐living secondary heterotrophs. Thus, these diatoms provide unique opportunities to study the evolutionary processes required to thrive without photosynthesis and independent of a resource‐providing host. They may also provide a chassis for reverse engineering photosynthesis in eukaryotic organisms. Here, we have developed a genetic transformation system for *N. putrida* using a biolistic approach. By leveraging genome and transcriptome data, we identified the nicotinamide adenine dinucleotide hydride (NADH)‐ubiquinone reductase complex 1 promoter as a robust candidate for driving transgene expression. Through Golden Gate Cloning, we engineered plasmids, including the selectable marker nourseothricin and the reporter *eGFP*. An evaluation of transformation efficiency confirmed the successful integration and expression of the transgenes. Fluorescence microscopy demonstrated the expression of *eGFP* in the transformed cell lines, which retained a growth phenotype similar to that of the wild type cells. Thus, our work in combination with the available genome and transcriptome of *N. putrida* enables reverse genetics with a free‐living secondary heterotroph.

**Abbreviations**
ATP: adenosine triphosphate
FACS: fluorescence‐activated cell sorting
LB: Luria‐Bertani (medium)
NADH: nicotinamide adenine dinucleotide hydride
Nat: nourseothricin
PCR: polymerase chain reaction
SDM: site‐directed mutagenesis

## INTRODUCTION

The evolutionary history of diatoms, single‐celled photosynthetic algae known for their nanopatterned silica cell walls, spans several hundred million years (Kooistra et al., 2007). Originating during the early Jurassic period, approximately 150–200 million years ago, diatoms have undergone a wide array of morphological and adaptive transitions (Kooistra et al., 2007).

They occupy a pivotal role especially in marine ecosystems, contributing significantly to annual global primary productivity (ca. 25%) and, therefore, to global biogeochemical cycles (Tréguer et al., 2018). Through photosynthesis, diatoms convert sunlight into chemical energy and organic carbon, accounting for over 40% of total marine primary productivity. Thus, their photosynthetic activity underscores their importance as part of the marine food web and the global carbon cycle.

However, the diversity and significance of diatoms extends beyond their role in aquatic photosynthesis. A notable example is the raphid pennate marine diatom *Nitzschia putrida* (Bacillariophyceae). This species secondarily lost photosynthesis and, therefore, evolved a heterotrophic mode of nutrition (Kamikawa et al., 2022). It is an osmotroph utilizing dissolved organic compounds as the primary source of energy. The transition from photosynthesis to obtaining nutrients through the consumption of organic matter marked a significant evolutionary shift. However, this transition in metabolic mode occurred multiple times within the class of diatoms (Frankovich et al., 2018; Kamikawa et al., 2015; Lewin & Lewin, 1967; Li & Volcani, 1987). The reason for why diatoms are prone to these major evolutionary transitions has yet to be revealed. Nevertheless, this deviation from the “norm” in diatom evolution presented itself as an exciting opportunity to study the consequences of the loss of photosynthesis, particularly in the context of a free‐living organism. Loss of photosynthesis in eukaryotic microorganisms has led to a diverse range of evolutionary adaptations, including osmotrophy, predation, and parasitism. Although many species have evolved the parasitic lifestyle, others have remained free‐living heterotrophs (McFadden & Yeh, 2017; Oborník, 2019).

Recent genomic studies have elucidated the molecular basis of *Nitzschia putrida*'s transition to heterotrophy. Unlike its photosynthetic relatives, *N. putrida* has lost all essential genes required for photosynthesis, including those encoding light‐harvesting fucoxanthin chlorophyll *a*/*c*‐binding proteins, photosystem I and II core subunits, and the Calvin‐Benson cycle enzymes, such as Rubisco (Kamikawa et al., 2022). Moreover, genes responsible for the biosynthesis of chlorophyll and plastoquinone have also been lost, rendering *N. putrida* fully non‐photosynthetic. Despite these losses, its plastid has not been entirely lost. Instead, it has retained genes essential for plastidial ATP synthesis, amino acid biosynthesis, heme production, and lipid metabolism, supporting its heterotrophic lifestyle (Kamikawa et al., 2022). Thus, these retained plastid functions highlight the ongoing metabolic necessity of the plastid, even in the absence of photosynthesis.

Because of these retained metabolic capacities of the plastid, *Nitzschia putrida* serves as an ideal model for studying the genetic, metabolic, and physiological changes that accompany a shift from photoautotrophy to heterotrophy. Unlike parasitic secondary heterotrophs, which rely on inter‐species dependencies, *N. putrida* provides a unique system for investigating how a free‐living eukaryote can survive without photosynthesis while maintaining a functional plastid. Furthermore, its reduced genome offers insights into the evolutionary constraints that dictate which metabolic pathways remain essential following the loss of photosynthesis. The presence of a plastid ATP synthase suggests the potential for reintroducing a light‐driven proton gradient, a concept that aligns with recent advancements in engineering alternative energy‐harvesting systems in heterotrophic eukaryotes (Peterson et al., 2024). By using *N. putrida*, future studies may explore strategies for reconstructing functional light‐driven energy systems in non‐photosynthetic plastids.

To meet this aim, we developed a plasmid suitable for biolistic transformation to introduce foreign genes into the genome of *Nitzschia putrida*. Using the experience gained from transformation systems previously developed for several diatom species such as *Thalassiosira pseudonana, Phaeodactylum tricornutum, Pseudo‐nitzschia arenysensis*, and *Fragilariopsis cylindrus* (Faktorová et al., 2020; Hopes et al., 2016; Poulsen & Kröger, 2005; Sabatino et al., 2015; Sprecher et al., 2023; Zaslavskaia et al., 2000), we tailored those systems for the unique needs of a heterotrophic diatom, which has a lifestyle more similar to yeast and *Escherichia coli*.

## MATERIALS AND METHODS

### Culture conditions for *N. putrida*

*Nitzschia putrida* (NIES‐4239) was obtained from the National Institute for Environmental Studies (NIES Collection, Japan). Cells were grown in 50% LB artificial seawater with a salinity of 16 prepared by mixing 20 g · L^−1^ Tropic Marin® artificial sea salt (Germany), 5 g · L^−1^ tryptone, and 5 g · L^−1^ yeast extract, supplemented with f/2 nutrients as per the National Centre for Marine Algae and Microbiota protocol (Guillard, 1975; Guillard & Ryther, 1962). For solid media, 0.8% agarose was added before autoclaving. An optional antibiotic mixture was used to remove bacterial contamination with 50 μg · mL^−1^ ampicillin, 1 μg · mL^−1^ gentamycin, 25 μg · mL^−1^ streptomycin, 1 μg · mL^−1^ chloramphenicol, and 10 μg · mL^−1^ ciprofloxacin. All cultures were grown at 20°C at 200 rpm to achieve higher cell concentration (Lewin & Lewin, 1967). Light is not required for growing *N. putrida*. Cell abundance over time was measured using a light microscope and 20x magnification. The growth rate per day (μ) during the exponential growth phase was calculated according to the following equation:

$$\mu \left(d^{-1}\right)=\frac{\ln N_{t2}-\ln N_{t1}}{∆t}$$

where *N*_*t*_ represents the cell density at the time *t*, and Δ*t* represents the time difference between the beginning and the end of the exponential growth phase.

### Testing growth on solid medium in the presence of antibiotics

To assess growth on solid medium, approximately 10^5^ cells were spread onto 0.8% and 1.6% agarose plates with f/2 medium and incubated under standard growth conditions. Colony formation was monitored for up to 14 days to see if colonies started to appear. To determine the lethal concentration of nourseothricin (Nat) and zeocin on *Nitzschia putrida*, wild‐type cell lines were incubated and monitored in liquid and solid f/2 medium supplemented with 50, 100, or 150 μg · mL^−1^ antibiotics for over 14 days (Sprecher et al., 2023; Zaslavskaia et al., 2000).

### Plasmid construction using Golden Gate cloning

Golden Gate cloning was performed based on the protocol outlined by Weber et al. (2011). Restriction enzyme BsaI and BpiI sites within target gene fragments were eliminated using the Q5 site‐directed mutagenesis (SDM) kit (NEB, United Kingdom). Plasmid DNA was extracted using the NEB Monarch Plasmid Miniprep kit. Golden Gate reactions for Level 1 and Level 2 assembly were adapted from the protocol by Hopes et al. (2016). Each 20 μL reaction was mixed with 40 fmol components, 100 units of BsaI or BpiI (NEB), 100 units of T4 DNA ligase, and 1x ligation buffer. Reactions were incubated at 37°C for 5 h, followed by 50°C for 5 min and 80°C for 10 min. Five microliters of the reaction mixture was added to 50 μL of NEB 10‐beta competent *Escherichia coli* DH10B (high efficiency) for further cloning and extraction.

#### Level 0 assembly

The endogenous promoter and terminator were selected based on transcriptome data from Kamikawa et al. (2022), specifically by selecting upstream and downstream sequences of the coding regions of genes with the highest expression levels. Following this strategy, we identified the NADH–ubiquinone reductase complex 1 MLRQ subunit (NADH; Kamikawa et al., 2022). Sequences were synthesized from Azenta (United States), with BpiI and BsaI sites subsequently removed by SDM. The enhanced green fluorescence protein (*eGFP*) gene from *Aequorea victoria* was amplified using Phusion DNA polymerase (NEB) and pUC:FCP:ShBle:FCP:eGFP (Faktorová et al., 2020) as a template with sense primer: 5′‐aggtctca*tacc*ATGGTGAGCAAGGGCGAGGA‐3′ and antisense primer: 5′‐aggtctca*gtat*TTACTTGTACAGCTCGTCC‐3′. The nourseothricin resistance gene (*nat*) was amplified from pICH47732:FCP:NAT by PCR using the sense primer: 5′ caaggtctcataccATGACCACTCTTGACGACACG‐3′ and antisense primer: 5′‐agtggtctcagtatTCAGGGGCAGGGCATGCTC‐3′, in which the uppercase letters are the original sequence, the lowercase letters are the additional sequence, and the underlined part is the enzyme recognition site (Hopes et al., 2016). All sequences were cloned into the pCR8/GW/TOPO vector (Thermo Fisher), with domestication of the BsaI and BpiI sites.

#### Level 1 assembly

The antibiotic resistance cassette was assembled with three L0 modules—NADH promoter, *nat*, and NADH terminator—into a L1 pICH47732 backbone, generating pICH47732:NADH:NAT. The Level 1 pICH47742 backbone was used to assemble the *eGFP* cassettes with the NADH promoter and NADH terminator, resulting in the plasmid pICH47742:NADH:eGFP.

#### Level 2 assembly

The L2 construct pNpNADH:NAT:eGFP was generated by combining the L1 modules pICH47732:NADH:NAT, pICH47742:NADH:eGFP with the L2E linker piCH41744, assembled into the L2 backbone pAGM4723. The construct underwent screening by SalI digestion and whole plasmid sequencing (Eurofins Genomics, Germany).

### Transformation and selection

Transformation of *Nitzchia putrida* cells was carried out using the Biolistic PDS‐1000/He particle delivery system (BIORAD, Hercules, CA, United States), following the protocols outlined by Poulsen et al. (2006) and Hopes et al. (2016). Transformations were performed in triplicate with the pNpNADH:NAT:eGFP; pICH47732:NADH:NAT; and water (negative control). Approximately 5 × 10^7^ cells in exponential phase were concentrated onto a 0.22‐μm filter by vacuum filtration and placed on 1.6% agarose plates containing f/2 medium. Plasmid DNA (3 μg) was coated onto 16 mg M10 (0.7 μm, Bio‐Rad) tungsten particles according to the manufacturer's protocol. Cells were then bombarded with a rupture disc of 1350 to 1550 psi and a flight distance of 7 cm. Following bombardment, cells were immediately resuspended in a 15‐mL standard culture medium for 24 h. Then ca. 10 million cells were transferred into three 20‐mL liquid medium vials containing 100 μg · mL^−1^ nourseothricin and incubated under optimal culture condition. Putatively transformed cells were collected after ca. 14 days.

To isolate single cell lines from the transformed mixture culture, fluorescence‐activated cell sorting (FACS) was performed. Before initiating the sorting process, cell suspension was prepared, ensuring that the cells were appropriately filtered by a 10‐μm filter and diluted to a concentration of approximately 100,000 cells · mL^−1^. The cell sorting was performed using a BD FACS Aria II Cell Sorter (Becton, Dickinson and Company, United States). Sorted cells were deposited into individual wells of a 96‐well plate with culture medium and 300 μg · mL^−1^ nourseothricin. Post‐sort verification of growth was conducted by a microplate reader using optical density (OD) at 600 nm and *eGFP* fluorescence.

### Screening clones and cultures

A 4‐mL culture was collected by centrifuging and the supernatant was removed. Cells were resuspended in 20 μL of lysis buffer (10% Triton X‐100, 20 mM Tris–HCl pH 8, 10 mM EDTA), stored at −80°C for 15 min, then incubated at 95°C for 10 min. Cells were screened for *eGFP* with primers 5′‐ATCATGGCCGACAAGCAGAA‐3′ (forward) and 5′‐TTACTTGTACAGCTCGTCCAT‐3′ (reverse), and *nat* with CTGGATGGGTCCTTCACCAC (forward) and TTGACGTTGGTGACCTCCAG (reverse) by polymerase chain reaction (PCR). The PCR products were sequenced by Eurofins (Germany).

### Analysis of gene expression by reverse transcription (RT‐PCR)

Eighty milliliters of cells from the exponential growth phase were harvested by centrifugation, and RNA was extracted using the Direct‐zol RNA Kits and RNA Clean & Concentrator Kit (Zymo, United States) according to the manufacturer's instructions. Reverse transcription used 0.2 μg of RNA with the SuperScript™ IV First‐Strand Synthesis System (Thermo Fisher, United States) following the manufacturer's instructions. Expressions of *eGFP* were analyzed using specific primer sets, with subsequent detection by RT‐PCR.

### Microscopy

Cell fluorescence images were obtained using an inverted Zeiss Observer 7 microscope and a 63× oil immersion objective (Carl Zeiss AG, Jena). The microscope settings were configured for multitrack and line acquisition to capture both brightfield and *eGFP* fluorescence simultaneously. Specifically, *eGFP* fluorescence was detected using a 550 nm bandpass filter.

## RESULTS

### Establishing a method for genetic transformation in *Nitzschia putrida*

To establish the genetic transformation system in *Nitzschia putrida*, we tailored the transformation protocols previously developed for diatoms such as *Thalassiosira pseudonana*, *Pseudo‐nitzschia arenysensis*, *Phaeodactylum tricornutum*, and *Fragilariopsis cylindrus* (Apt et al., 1996; Poulsen et al., 2006; Poulsen & Kröger, 2005; Sabatino et al., 2015; Sprecher et al., 2023; Zaslavskaia et al., 2000). To test the growth of *N. putrida* on solid agarose, we utilized plates with two different concentrations: 0.8% and 1.6% of agarose. After 7 days of cultivation, *N. putrida* exhibited significant growth on 0.8% agarose, characterized by denser cell populations compared with other concentrations tested. Additionally, we tested the sensitivity of *N. putrida* to antibiotics by incubating cells with 50, 100, and 150 μg · mL^−1^ of nourseothricin and zeocin in liquid medium for 10 days. Results indicated that concentration of 100 and 150 μg · mL^−1^ nourseothricin and zeocin effectively inhibited cell growth, respectively (Table 1). However, due to the cell toxicity of zeocin (van Peer et al., 2009), we opted to use 100 μg · mL^−1^ of nourseothricin for our transformation system.

**TABLE 1:** Antibiotic sensitivity of *Nitzschia putrida*.

Cell concentration	Antibiotics concentration (μg · mL^−1^)		
	50	100	150
Nat 10^5^ cells · mL (liquid)	−	−	−
Nat 10^6^ cells · mL(liquid)	+	−	−
Nat 5 × 10^7^ cells (solid)	+	−	−
Zeocin 10^5^ cells · mL (liquid)	−	−	−
Zeocin 10^6^ cells · mL (liquid)	−	−	−
Zeocin 5 × 10^7^ cells (solid)	−	−	−

*Note*: “+” denoting the presence of living cells in flasks after 10 days of selection, and “−” indicating their absence. Abbreviation: Nat, Nourseothricin.

**FIGURE 1:**
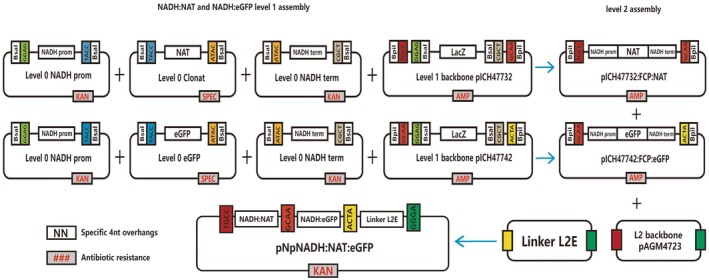
Overview of Golden Gate cloning assembly for pICH47732:NADH:NAT and pNpNADH:NAT:EGFP used in transforming *Nitzschia putrida*. Level 0 (L0) modules are created by PCR and then cloned into pCR8/GW/TOPO vectors. Construction of the level 1 (L1) and level 2 (L2) modules uses BsaI and BpiI restriction enzymes. Key components include the promoter (NADHpr) and terminator (NADHt) of the NADH–ubiquinone reductase complex 1 MLRQ subunit, ampicillin resistance gene (AmpR), kanamycin resistance gene (KanR), and spectinomycin resistance gene (SPEC). See the main text for additional abbreviations and details. Shared overhangs were indicated in different colors.

As a heterotrophic diatom, *Nitzschia putrida* lacks all major photosynthesis genes such as genes encoding fucoxanthin chlorophyll *a*/*c* binding proteins, commonly used for driving transgene expression (Fischer et al., 1999; Kilian & Kroth, 2005; Poulsen & Kröger, 2005; Zaslavskaia et al., 2000). Therefore, to drive efficient expression of the nourseothricin marker gene and the reporter gene, we checked the gene expression data of *N. putrida* (Kamikawa et al., 2022) and identified several endogenous genes with high expression (Table 2). Among those, the gene encoding the NADH–ubiquinone reductase complex 1, a key component of the mitochondrial respiratory chain, exhibited the highest expression and, therefore, likely possessed a strong promoter (Almutairi, 2022; Cardol, 2011; Table 2). Consequently, we selected the promoter from the NADH–ubiquinone reductase complex 1 gene to drive the expression of our transgenes. To construct an expression vector based on the NADH promoter for *N. putrida*, we synthesized 1000 bp upstream of the ATG start codon and 831 bp downstream of the NADH–ubiquinone reductase complex 1 gene stop codon. Subsequently, the plasmid pICH47732:NADH:NAT was constructed using Golden Gate Cloning (Figure 1). *Nitzschia putrida* cells were cultured in a liquid medium for 2 days to reach the mid/end of the exponential phase before bombardment. Cells were immediately transferred into non‐selective medium for 24 h and then returned to selective liquid medium.

**TABLE 2:** The 10 highest expressed genes in *Nitzschia putrida* under different growth conditions.

ID	Transcripts per million	Number of reads	Predicted functions
NAA15P02220.m1	40,741.12	531,595.41	NADH–ubiquinone reductase complex 1 MLRQ subunit
NAA02P08450.m1	13,828.98	129,286.20	Small subunit ribosomal protein S28e
NAA41P00270.m1	12,104.54	279,594.59	Cytochrome c
NAA03P04550.m1	10,733.23	205,861.61	Ribosomal protein L41
NAA18P02090.m1	7100.36	127,175.67	60S ribosomal protein L37
NAA14P02710.m1	6872.14	214,966.96	Ribosomal protein L44e
NAA07P03020.m1	6236.76	19,5091.68	60s acidic ribosomal protein
NAA42P00920.m1	6027.24	222,393.81	Cytochrome c oxidase subunit 6B
NAA03P01270.m1	5789.20	487,003.74	Acetate transporter
NAA07P01730.m1	5782.82	141,314.63	40S ribosomal protein S30

After ca. 14 days, cells were first observed under selective growth conditions. These nourseothricin resistant cells were collected from independent flasks by centrifugation, and the presence of *nat* was confirmed by PCR (Figure S1a). We successfully recovered eight cultures of *Nitzschia putrida* with the nourseothricin resistance phenotype from nine flasks (Table S1). Additionally, we attempted the selection of resistant strains using agarose plates by spreading 5 × 10^6^ cells on selective plates 24 h after bombardment. However, due to the significant cell motility, no individual colonies were formed; instead, a lawn of cells was observed homogeneously covering the surface of the plates (Figure S2). Consequently, liquid medium was chosen for the first selection of the transformed cells and subsequent cultivation of the genetically modified cultures. Fluorescence‐activated cell sorting was then used to isolate single cell lines of the transformed strains.

### Expression of exogenous proteins in *Nitzschia putrida*

To evaluate the feasibility of exogenous protein expression, we selected the reporter *eGFP*, which is absent in *Nitzschia putrida*. Codon bias analysis of the *eGFP* gene was conducted using MEGA11 (Tamura et al., 2021). To overexpress *eGFP* in this diatom, we assembled two overexpression plasmids, namely pNpNADH:NAT:eGFP through Golden Gate cloning with pICH47732:NADH:NAT (Figure 1). As a consequence, six *N. putrida* strains were recovered from nine flaks after transformation. Single cell lines of the *eGFP* overexpressed strains were isolated using FACS (Figure S3). We screened six *N. putrida* strains with the *eGFP* gene from their respective three bombardments and sequenced the purified PCR products for confirmation of the presence of the *eGFP* gene (Figure S4). As a further confirmation of the translation of *eGFP*, we extracted RNA and performed reverse transcription. The RT‐PCR results revealed the presence of *eGFP* in the transformed cells, whereas there was no amplification in the wild type and our negative control (Figure S1b).

**FIGURE 2:**
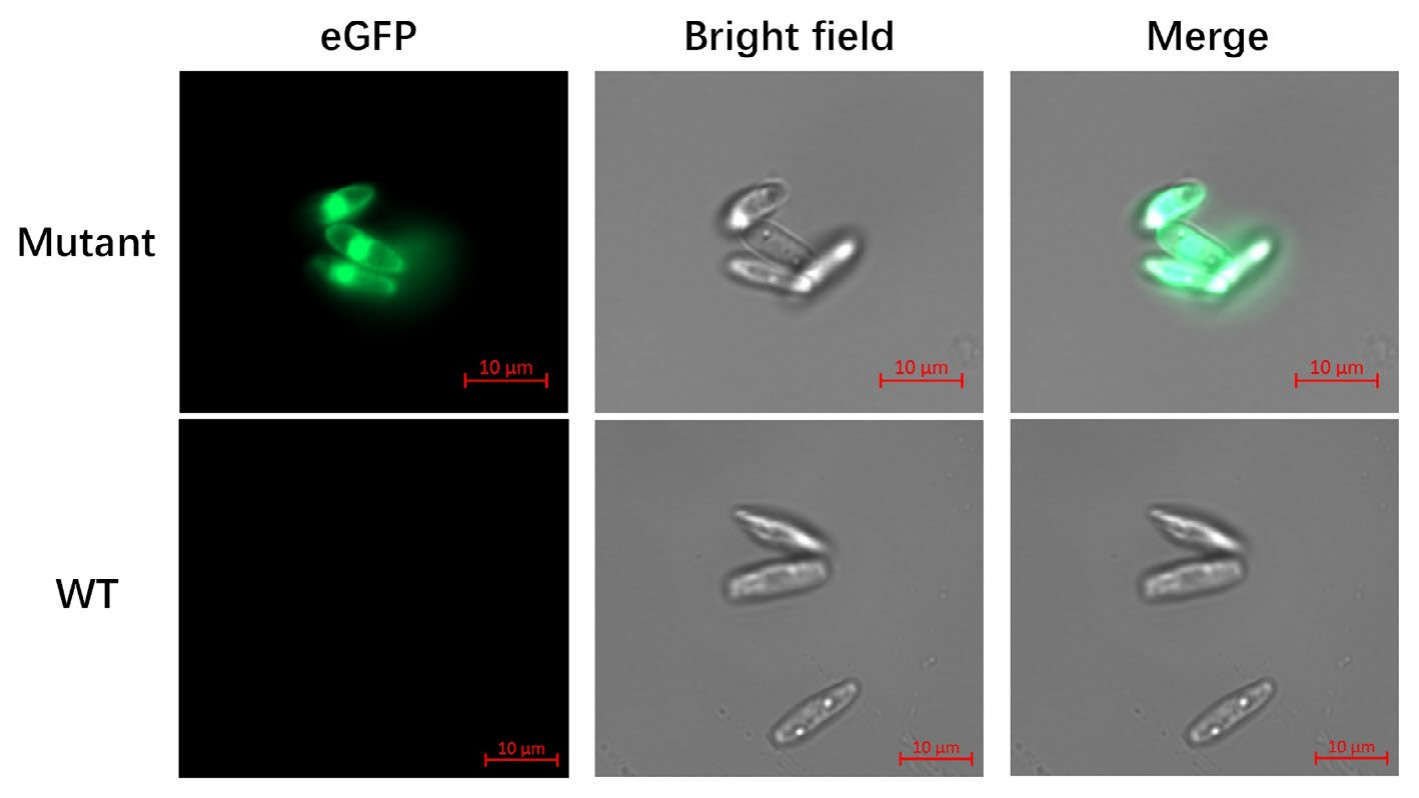
Fluorescence microscopy of wild‐type (WT) and enhanced green fluorescence protein (*eGFP*) overexpressing *Nitzschia putrida* single cell line strains. Shown are bright field and *eGFP* images, and a merge of both. Scale bars, 10 μm.

**FIGURE 3:**
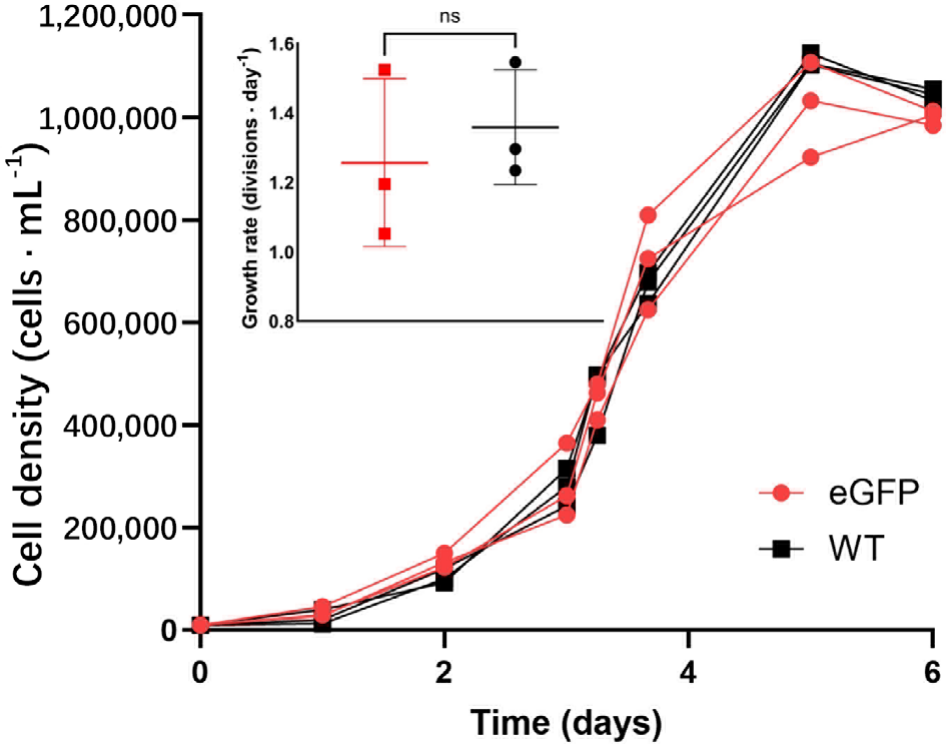
Growth of wild‐type (WT, black) and *eGFP* overexpressing (*eGFP*, red) *Nitzschia putrida* strains in control medium with three replicates. Growth rates indicate no significant difference between the knock‐in mutant strain and the wild type. Error bars denote *SD*.

Using fluorescence microscopy, we confirmed the expression of *eGFP*. Significant fluorescence was observed in all six *eGFP* mutant strains, whereas the wild type showed no fluorescence (Figure 2). After 4 months of cultivation in liquid medium in the laboratory, growth experiments were conducted with the transgenic strains to see if transformation had a significant impact on their phenotype. At least under optimal growth conditions (e.g., nutrient repletion), the growth of the transgenic cell lines showed no significant difference to the wild type (Figure 3), suggesting that the established transformation system for *Nitzschia putrida* did not affect cell division.

## DISCUSSION

To the best of our knowledge, this paper reports on the first stable genetic transformation system of a heterotrophic diatom. Compared to other diatoms, the absence of highly expressed photosynthetic genes such as fucoxanthin chlorophyll *a*/*c*‐binding proteins (fcp), which are commonly used in many diatom transformation systems (Li et al., 2025), means it is required to identify suitable promoters and terminators from other highly expressed endogenous genes. The gene of our choice was the NADH–ubiquinone reductase complex 1. However, it is likely that promoters from other highly and constitutively expressed genes such as cytochrome c and ribosomal protein L41 work equally well in *Nitzschia putrida* (Table 2).

Due to the significant motility of *Nitzschia putrida* cells on surfaces (Figure S2), a liquid medium for selection was the preferred choice. However, an issue with selection in liquid medium is that many cells with potentially different numbers of plasmids integrated into their genomes contribute to the growing culture. Thus, the population of transformed cells is likely genetically diverse, yet they all share the presence of the cloned transgenes. Thus, to work with a genetically identical population of cells, we applied FACS to the cultures we had generated under selective conditions. Using *eGFP* expression and cell‐sorting into 96‐well plates to produce different clonal lineages allowed us to work with biological replicates that were genetically distinct (Hu et al., 2016; Marie et al., 2017; Sinigalliano et al., 2009).

Taken together, the successful establishment of a genetic transformation system in *Nitzschia putrida* opens new avenues for molecular research in free‐living secondary heterotrophs by addressing fundamental questions about their evolution to reverse engineering of photosynthesis in a eukaryotic host. The latter might not only improve the yield of any desired high‐value product synthesized by these organisms (e.g., essential polyunsaturated fatty acids such as eicosapentaenoic acids; Kamikawa et al., 2022), but it might also shed new light on how to design a minimal photosynthetic chloroplast.

## AUTHOR CONTRIBUTIONS

**Longji Deng:** Data curation (equal); formal analysis (equal); methodology (equal); writing – original draft (equal). **Yixuan Li:** Methodology (equal); writing – review and editing (equal). **Yasuhiro Tanizawa:** Methodology (equal); writing – review and editing (equal). **Yasukazu Nakamura:** Methodology (equal); writing – review and editing (equal). **Ryoma Kamikawa:** Project administration (equal); writing – review and editing (equal). **Amanda Hopes:** Methodology (equal); writing – review and editing (equal). **Thomas Mock:** Conceptualization (equal); data curation (equal); methodology (equal); project administration (equal); writing – review and editing (equal).

## Supporting information


**Figure S1.** Screening by PCR amplification of gDNA and cDNA with separate primer sets to amplify *nat*, and *eGFP* (eG). “WT,” wild type; “N,” PCR negative control replaced DNA with water. (a) Amplification of *nat* from two wild‐type and four overexpressing strains. Target *nat* production length 374 bp. Different lanes loaded with different strains. (b) RT‐PCRof *eGFP* from wild‐type and overexpressing strains. Target *eGFP* production length 264 bp. “E,” *eGFP* gene; “N,” negative control replaced transcriptase by water.

**Figure S2.** Agarose plates with *Nitzschia putrida* cells. The diatom colonies are outlined with a red line. (A) Cells growing on f/2 selective medium. (B) Wild‐type cells growing with antibiotics as a negative control.

**Figure S3.** Flow cytometry analysis of *eGFP*‐expressing diatom strains. Fluorescence‐activated cell sorting (FACS) was used to evaluate the expression of enhanced green fluorescent protein (*eGFP*) in transgenic diatom strains. The histogram displays the fluorescence intensity (FL1‐A) of the *eGFP* strains divided into fluorescence signal and no fluorescence groups.

**Figure S4.** PCR screening of *eGFP* integration in transgenic strains. Genomic DNA (gDNA) from wild‐type (WT) and *eGFP*‐expressing (eg) strains was amplified by PCR using the primers: forward, 5′‐AGGACGACGGCAACTACAAG‐3′ and reverse, 5′‐TTCTGCTTGTCGGCCATGAT‐3′. The expected amplicon size for *eGFP* is 172 bp. Different lanes represent individual strains. Detection of the *eGFP*‐specific band indicates successful integration of the transgene.

**Table S1.** Summary of successful transformation experiments in *Nitzschia putrida*. Each was performed with three bombardments.
