# Supplementary figures and images for: A genetic transformation system for the heterotrophic diatom Nitzschia putrida (Bacillariophyceae)
Source: J Phycol. 2025 Aug 14;61(5):1116–23. doi: 10.1111/jpy.70070 (PMC12547632; doi:10.1111/jpy.70070)

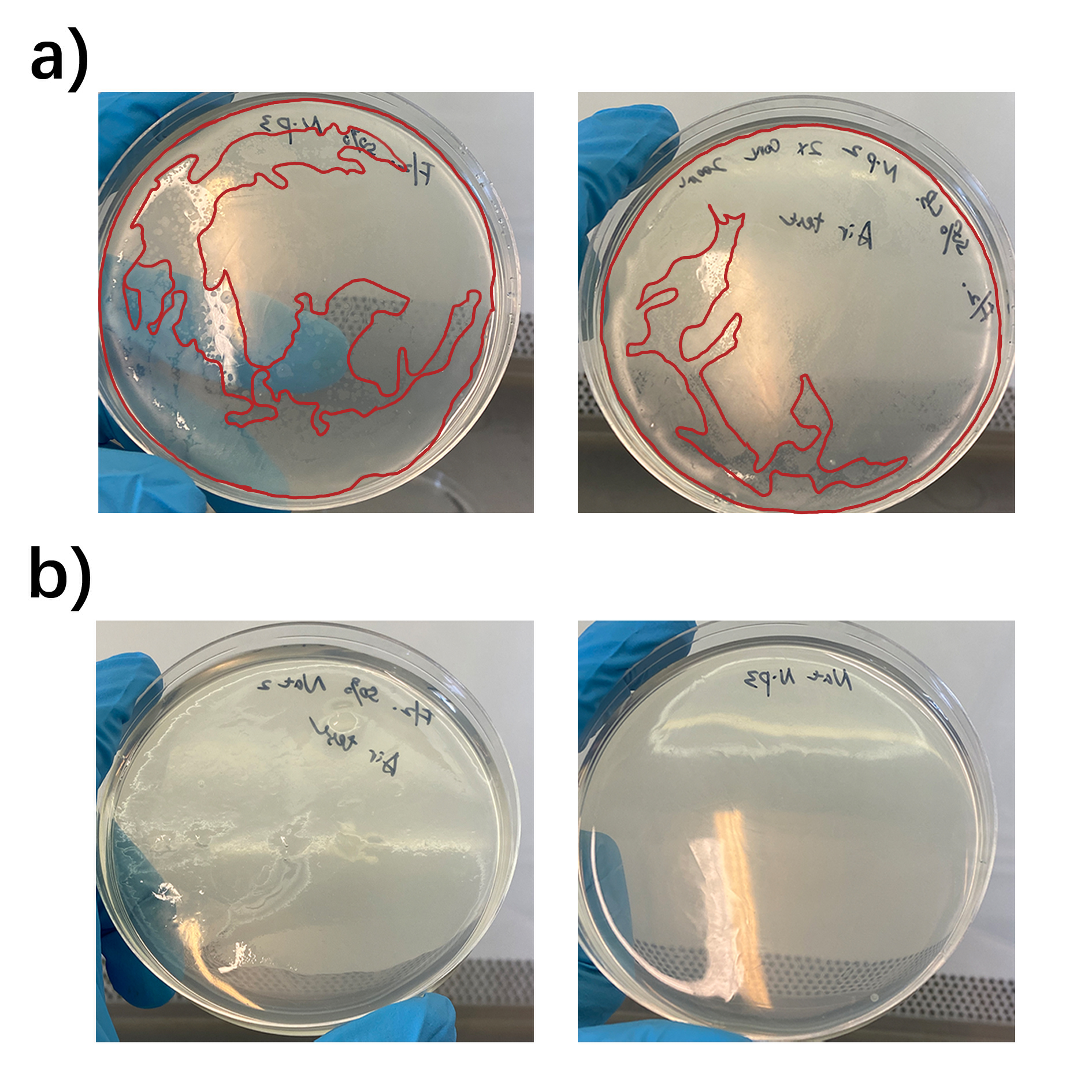

Supplement: Supplementary file 2 — Figure S2. Agarose plates with Nitzschia putrida cells. The diatom colonies are outlined with a red line. (A) Cells growing on f/2 selective medium. (B) Wild‐type cells growing with antibiotics as a negative control. [file JPY-61-1116-s003.png]

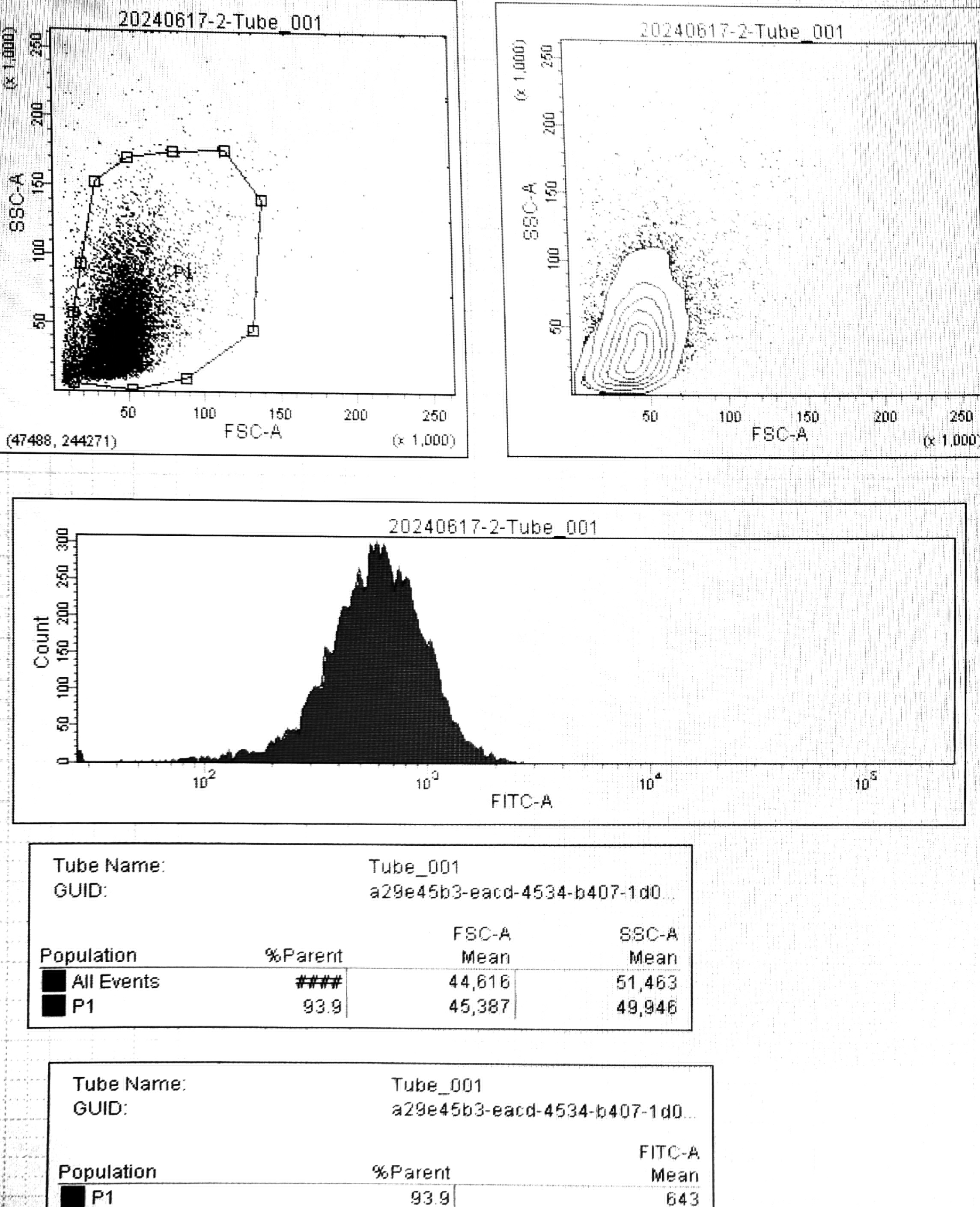

Supplement: Supplementary file 3 — Figure S3. Flow cytometry analysis of eGFP‐expressing diatom strains. Fluorescence‐activated cell sorting (FACS) was used to evaluate the expression of enhanced green fluorescent protein (eGFP) in transgenic diatom strains. The histogram displays the fluorescence intensity (FL1‐A) of the eGFP strains divided into fluorescence signal and no fluorescence groups. [file JPY-61-1116-s002.tif]
