# Supplementary material for: A genetic transformation system for the heterotrophic diatom Nitzschia putrida (Bacillariophyceae)
Source: J Phycol. 2025 Aug 14;61(5):1116–23. doi: 10.1111/jpy.70070 (PMC12547632; doi:10.1111/jpy.70070)
Supplement: Supplementary file 5 — Table S1. Summary of successful transformation experiments in Nitzschia putrida. Each was performed with three bombardments. [file JPY-61-1116-s005.docx]

Table S1. Summary of successful transformation experiments with *N. putrida*.

| Plasmid | Number of flasks with bombarded cell cultures | Flasks with cells positive for target transgene |
| --- | --- | --- |
| pICH47732:NADH:NAT | 9 | 8 |
| pNpNADH:NAT:eGFP | 9 | 6 |
